# Contested involvement of family members in service allocation processes in long-term care: a qualitative study

**DOI:** 10.1186/s12877-026-07265-5

**Published:** 2026-03-03

**Authors:** Ann Katrin Blø Pedersen, Marianne Sundlisæter Skinner, Hege Skundberg-Kletthagen, Maren Sogstad

**Affiliations:** Centre for Care Research, Department of Health Sciences in Gjøvik, Faculty of Medicine and Health Sciences, NTNU – Norwegian University of Science and Technology, P.O. Box 191, Teknologivegen 22, Gjøvik, 2802 Norway

**Keywords:** Needs assessment, Purchaser-provider, Collaboration, Family caregivers, Older adults

## Abstract

**Background:**

Population ageing, as the predominant demographic trend in many Western countries, has prompted governments to seek novel approaches to long-term care provision. Increased collaboration between formal and informal caregivers in service planning and provision is one solution advocated by governments across Europe and other regions. This qualitative study explored service allocators’ experiences with involving family members in the assessment and allocation of long-term care services for older adults in Norway.

**Methods:**

Using content analysis, we analysed data from semi-structured individual interviews (*n* = 9), focus group interviews (*n* = 2), and hours of direct observation (*n* = 165), all with or of service allocators tasked with assessing needs and assigning long-term care services to older adults.

**Results:**

The overarching theme identified was Balancing Family Involvement: Legal Rights, Efficiency, and Welfare. The themes identified were (i) Conflict in Involving Family Members in Caring, (ii) Efficient Involvement of Family Carers in Needs Assessment, and (iii) Support: Preserving the Family Caregiver Role.

**Conclusions:**

By prioritising system efficiency over family members’ values, New Public Management principles influence and obstruct the dynamics and effectiveness of family members’ involvement in the long-term care allocation process. To improve and enhance the involvement of family members, a paradigm shift towards a more family-oriented approach within the long-term care system is needed.

**Trial registration:**

Not applicable.

**Supplementary Information:**

The online version contains supplementary material available at 10.1186/s12877-026-07265-5.

## Background

Ageing populations pose an important global challenge to long-term care systems [1, 2]. Increased care needs with age will increase the number of citizens needing long-term care. Older adults requiring long-term care may apply for a range of services and support options, including home-based care, assisted living arrangements, nursing home placement, day-care facilities, personal assistance, and emergency response systems such as social alarms.

Internationally, there are considerable gaps in financial coverage of and access to long-term care services [3]. Therefore, the demand for informal care is expected to rise [4]. In Europe, the most frequent form of care provided to dependent older adults is informal care by family members [5]. Family caregivers, also known as informal caregivers, assist in reducing the costs associated with public long-term care provision for older adults [6]. A family caregiver is a person who provides care for a relative with care needs caused by long-term illness, disablement or age [7]. He or she can be a spouse, son or daughter performing care tasks and actions that significantly affect the recipient’s health, well-being and/or daily life [8]. Empirical studies suggest that managers and formal caregivers recognise family members as invaluable contributors to care delivery [9] and rely heavily on family members’ contributions to care provision in the home [10, 11]. Nevertheless, the service recipient’s needs and interests are central and guide family involvement, though divergent perceptions between users and family members may complicate this process [12–14]. Additionally, society’s reliance on family caregivers has its own challenges and disadvantages. Family members who provide care for older adults with disabilities experience considerable caring burden and stress related to their role as caregivers [15, 16]. Caregivers’ health is a crucial public policy concern in several Organisation for Economic Co-operation and Development (OECD) countries [17] and has led to the implementation of measures aimed at facilitating improved access to information, counselling, training, and respite for informal caregivers [6].

Research shows that informal caregivers want to be involved in the care of their family members and that they appreciate that formal caregivers acknowledge their contributions [18–20]. However, there is limited research into specific mechanisms that can improve the engagement and experience of families in caring for family members in long-term care settings [21, 22] and to what extent service allocators in long-term care involve family members in the assessment and allocation of services. Norway is an example of a universal welfare state where there has been increasing political attention to family caregiving as a means of achieving welfare goals in recent years [23]. The aim of this study was to explore service allocators’ experiences involving family members in the needs assessment and allocation of long-term care services to older adults in Norway. While the study is rooted in the context of Norwegian long-term care, the expanding global emphasis on the involvement of informal caregivers in long-term care planning and provision suggests that the findings and discussion are relevant to scholars, policy-makers, and other stakeholders in other long-term care systems.

### The Norwegian context

In Norway, municipalities are the administrative local government units responsible for delivering long-term care services to the population [24]. Long-term care services constitute one of the municipality’s primary responsibilities and are overseen by a head of long-term care services in each municipality. According to guidelines from the Norwegian Directorate of Health [25], municipalities are required to establish strategies for involving family members within the long-term care system. New Public Management (NPM) is a reform ideology that has influenced public sector governance in Norway, as in many Western countries, including the organisation and allocation of health care services [26–30]. Rooted in free-market principles, NPM seeks to reduce the size and scope of government through decentralisation, privatisation, and the removal of bureaucratic structures [31]. It emphasises efficiency, accountability, and performance measurement—values typically associated with the private sector. Within this framework, citizens are reframed as “customers,” or “consumers”, and public servants are positioned as “managers” responsible for delivering services in a cost-effective manner [32]. While NPM aims to improve service delivery, its emphasis on market logic and managerial efficiency has raised concerns about its suitability in sectors such as long-term care, where complex needs and relational aspects of care may be poorly served by performance-driven models [29, 33]. Following ideas from NPM, many Norwegian municipalities have implemented a purchaser-provider model that splits the allocation and provision of long-term care services into two separate units [29, 30, 34]. The purchaser-provider model draws on standardisation and formal requirements to guide service allocation. Furthermore, it builds on formal contracts that guide the relationship between long-term care providers and care receivers [29]. When a care need arises, the person applies for care services from the local municipality, for example, nursing home placement or home care services. In the first step, the service allocators at the allocation unit assess care needs and decide on the appropriate service level and type based on specific criteria. For example, nursing home placement is typical based on criteria such as a high need for medical care and/or assistance with activities of daily living [35]. They gather and provide information before specifying contracts and ordering services. Usually, the initial assessment is performed through a conversation conducted in the care applicant’s home, preferably with family members present. In the second step, the provider unit is responsible for delivering services specified by the service allocators [29].

In Norway, the regulatory framework governing the interaction between formal and informal caregivers is outlined by the Patient and User Rights Act [36] and the Municipal Health and Care Services Act [37]. The former act states that long-term care services must be directed and customised to meet the specific requirements of the care recipient. The latter act stipulates that the municipality is responsible for long-term care for (older) adults, thereby establishing that care contributions from family members are voluntary. Consequently, formal caregivers cannot assume or anticipate contributions from family members to the provision of care, at least not within the scope of legislative directives [38]. In the Norwegian system, service allocators are tasked with assessing individuals’ needs for long-term care, determining appropriate long-term care services, and prioritising access in contexts of resource scarcity [39]. Thus, service allocators have a “gatekeeping role” in relation to citizens’ access to welfare benefits. A central challenge lies in negotiating competing demands across diverse user groups—for example, balancing the needs of younger versus older individuals, those with cognitive impairments such as dementia, and persons requiring rehabilitative as opposed to long-term supportive care [40–42]. These decisions necessitate careful deliberation regarding what qualifies as essential assistance, the appropriate distribution of care responsibilities between public authorities and private actors (e.g. families), and the anticipated consequences of interventions over time [43]. While service allocators are required to adhere to established regulations and legal frameworks, they have historically held a relatively high degree of autonomy in their professional practice [44, 45]. In Norway, the majority of service allocators are nurses, with the remaining comprising allied health and social care professionals (e.g. physiotherapists, occupational therapists, and social workers) [46]. This indicates a predominance of health professionals in service allocation roles. Service allocators must reconcile professional obligations to individual care with systemic imperatives for cost-efficiency. Balancing professional, political, and administrative logics presents an ongoing challenge within an increasingly pressured health and long-term care system [47].

In universal welfare states such as Norway, formal long-term care professionals often deliver intensive and highly skilled care services, such as medical care and nursing home placements. On the other hand, informal caregivers typically assume responsibility for care activities of a nonskilled nature [48, 49], such as practical assistance. The financing of long-term care services predominantly emanates from public sources via general taxation, with allocation contingent upon assessed needs [50]. The Norwegian long-term care services include respite encompassing various interventions that temporarily alleviate the caregiving burden for family members. The primary forms of respite include day-care services, in-home respite, and temporary institutional care stays within long-term care facilities [50]. The only social benefit in Norway for family long-term carers consists of a care wage (*omsorgslønn*), which is a form of paid compensation. This system, however, is usually limited to high-burden care; therefore, only a small amount of the care work carried out by family members is paid work [23].

## Methods

### Study design and setting

This qualitative study is empirically grounded in the Norwegian context. We combined data from individual interviews, focus groups and direct observations to explore service allocators’ considerations and prioritisation processes in depth [51]. Integrating these varied methods serves to increase the volume of data while also yielding richer, contextually grounded insights [52]. Norwegian municipalities vary in geographical size, how they organise long-term care, and how many citizens they serve [35]. To facilitate variation across settings, one small (< 5000 citizens), one medium-sized (5000–34999 citizens) and one large municipality (> 35000 citizens) were included in the study. Two of the included municipalities had a purchaser-provider model, while the third had a split model where the informants worked half the time as service allocators and the other half as service providers. The study is reported in accordance with the COnsolidated criteria for REporting Qualitative research (COREQ) checklist (see Supplementary Material 1).

### Recruitment and participants

To recruit the service allocators, an invitation to participate in the study was sent to the heads of long-term care services in the three municipalities. Staff who assessed and allocated long-term care services for adults 65 years of age and above were included in the study. Nine service allocators were recruited by their leaders for individual interviews: four from the large municipality, three from the medium-sized municipality and two from the small municipality. The “information power” of the study was enhanced by the researcher recruiting three additional participants from the medium-sized municipality and one more participant from the large municipality for focus groups and direct observation of daily practice. A total of thirteen service allocators were informed about the study and consented to participate. Information power refers to the richness of the data and the potential of the sample to achieve the aim of the study [53]. All thirteen participants were women. Nine participants were nurses, two were physiotherapists, and two were occupational therapists. There was no social workers or social service providers involved in the allocation process in any of these municipalities during the data collection period. No prior interaction or relationship was formed with any of the participants before the initiation of the study.

### Data

Data collection took place between August and November 2020.

#### The interviews

A total of nine semi-structured individual face-to-face interviews and two focus groups were conducted. The first author, a female PhD candidate holding a master’s degree in health sciences, conducted every interview at the service allocators’ workplaces. At the outset of each interview, she gave a short introduction to her background, including her academic credentials, epistemological assumptions, research rationale, and specific interests in the research topic. Individual interviews lasted between 45 min and one hour, while the focus groups extended from 60 to 90 min. The study was part of a project where the overall aim was to study service allocators’ experiences with considerations, dilemmas, and collaboration in the needs assessment process and allocation of services to older adults, and where two interview guides was developed based on current literature on needs assessment and priority setting in service allocation (see Supplementary Material 2). The interview guides did not directly address the involvement of family members in the allocation process. However, the interviewees themselves addressed the topic (unprompted) in the interviews, providing the impetus to analyse this in particular in this study. Although other informal caregivers may also be relevant to this research, family members were chosen, as they were the subjects mainly referred to by service allocators in the study. In this study family members involve spouses, sons and daughters and other family members such as sons- and daughters-in-law. The focus groups took place after the completion of individual interviews and direct observations in each municipality, and included five and six participants, respectively. The purpose of the focus groups was to address dilemmas that had emerged as prominent during the individual interviews and observations.

#### The observations

The study involved the direct observation of weekly allocation meetings, at the allocation units and/or at the nursing home units, wherein the assessment of services to individual users was deliberated. These meetings primarily focused on determining which older adults should receive nursing home stays. Participants in these meetings comprised either service allocators only or service allocators and long-term care professionals such as nurses, physiotherapists, occupational therapists, and nursing home leaders. Attendance varied depending on the meeting agenda and the need for specific insight and expertise. The first author conducted observations of fourteen such meetings across the municipalities, each lasting between 60 and 90 min. To gain a deeper understanding of the priority setting and reasoning for service allocation, the observations focused on what meeting participants emphasised during discussions about the allocation of long-term care services for older adults. Additionally, informal conversations were conducted with the thirteen service allocators before and after the meetings, asking questions to clarify issues arising in the observations [51, 54]. A total of 165 h of observation were dedicated to the daily practices of service allocators, including the allocation meetings and home visits. A total of 6 home visits were observed, some with and some without family members present. Before these home visits, involving service recipients, the service allocators informed the individuals and any family members present of the researcher’s presence and the purpose of the project, and obtained their informed consent prior to the meeting. This consent was provided verbally. Field notes were taken, and any information that could identify patients was omitted to preserve confidentiality.

### Disclosures and ethics

The study adhered to the ethical standards of the 1964 Declaration of Helsinki and its subsequent amendments. Ethical approval was granted by the Regional Committees for Medical and Health Research Ethics, REK North (2020/111946), and the Norwegian Centre for Research Data (NSD/Sikt; reference number 693007). Prior written informed consent was obtained from all study participants, who were informed about their right to withdraw from the study at any time. The written materials outlined key aspects such as the purpose of the study, procedures for handling sensitive data, participants’ rights, and relevant contact details. Confidentiality was maintained throughout the study, including in the reporting of the findings.

### Coding and data analysis

All the interviews were audio-recorded and transcribed verbatim. All the interviews and field notes were closely read, and these texts were systematically condensed into meaningful units [55] and codes related to the service allocators’ involvement of family members when assessing and allocating services for older adults. NVivo 20 software was used to organise, review, and analyse meaning units and codes.

Categories and themes were then identified through qualitative content analysis [55]. While the individual interviews served as the primary data source in the analysis, supplementary information was added from the field notes and focus group discussions to deepen the researchers’ understanding of the context and the service allocators’ involvement of family members in needs assessment and the allocation of long-term care services. The data analysis started with coding of the individual interviews, which were followed by the focus groups and then the field notes.

To ensure consistency and depth, the research team held several meetings to develop and refine codes, categories and themes. We also scrutinised the data for any inconsistencies across the different types of sources; however, no such discrepancies were identified. For instance, interviewees’ claims that they consider family members during needs assessments were corroborated by observations made during allocation meetings. The purpose of variation in data sources was to broaden the data (based on the experience that both organisation and practice might vary dependent on the municipal size) and not to compare between municipalities or settings. Nevertheless, the impression from the data was that the practice was (relatively) consistent regardless of profession, organisation and municipality.

The analysis resulted in three themes addressing the involvement of family members in the allocation process (see Table 1), and the three themes were further abstracted into a main theme: Balancing Family Involvement: Legal Rights, Efficiency, and Welfare. The first author conducted the first steps of the analysis, and all three researchers were involved in determining the themes and main theme. Finally, all the authors critically reviewed the themes and reread the codes and categories, ensuring a balance of closeness to and analytical distance from the data [56] and a shared understanding and interpretation of the results.

**Table 1:** Main theme and themes of the involvement of family members in service allocation processes

Balancing Family Involvement: Legal Rights, Efficiency, and Welfare		
Conflict in Involving Family Members in Caring	Efficient Involvement of Family Carers in Needs Assessment	Support: Preserving the Family Caregiver Role
Legal requirements challenge the balance between the voluntary nature of the family caregiver role and service allocators’ expectations of their care contributions.	Involving family members to gather information about available services to set realistic expectations.	Involving family members to support and preserve the family caregiver role to delay escalation of service use.

## Results

The overarching theme identified was Balancing Family Involvement: Legal Rights, Efficiency, and Welfare. The themes identified were (i) Conflict in Involving Family Members in Caring, (ii) Efficient Involvement of Family Carers in Needs Assessment, and (iii) Support: Preserving the Family Caregiver Role (see Table 1).

**Fig. 1:**
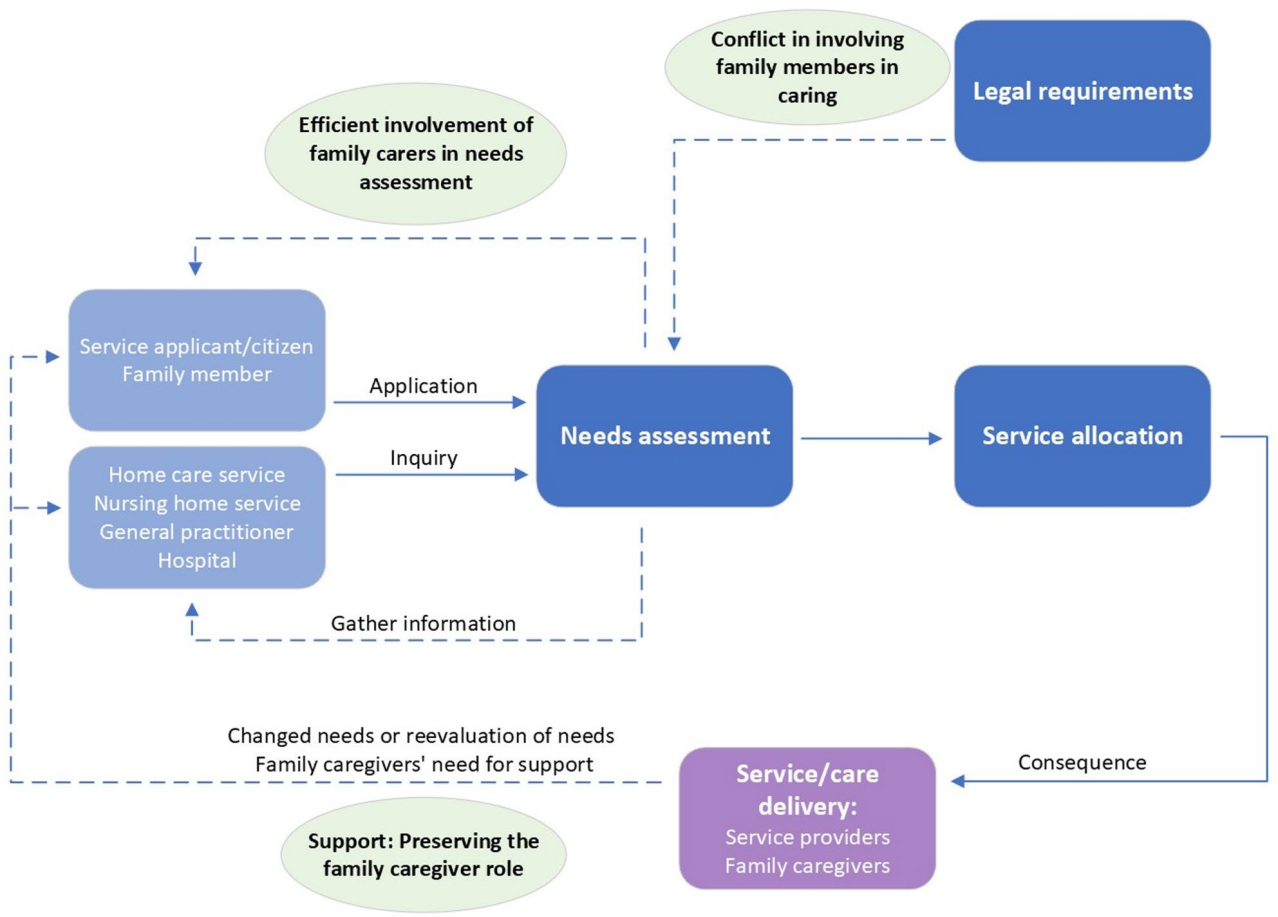
Involvement of family members in service allocation processes

The three themes came into play in different parts of the service allocation process, as illustrated in Fig. 1. Theme one was about how the legal requirements affected the involvement of family members, causing service allocators to experience a conflict in balancing their expectations of family care contributions with the principle of voluntarism. Theme two covered the involvement strategies service allocators used in the first stages of needs assessment, which primarily centred on information exchange with family members, leading to challenges in balancing efficiency and family involvement. The final theme concerned the service allocators’ strategies to preserve family caregivers in their caregiver roles, focusing on their need for support. This was typical in cases where family members had a high caregiver burden over time and/or there was a change in the care recipient’s health status, which necessitated a reassessment of needs (caregivers’ and/or care recipient’s needs). The overarching challenge was to balance family involvement in the care of older adults without compromising the safety of care recipients or the welfare of family caregivers.

### Conflict in involving family members in caring

Service allocators frequently deliberated about the role of family members in caring for older adults, recognising the willingness of several family members to actively contribute to meeting service recipients’ care needs. However, the municipality is legally responsible for covering citizens’ care needs. The importance of assistance and the involvement of family members were emphasised by service allocators. However, striking a balance between the voluntary nature of the family caregiver role and imposition by service allocators posed a conflict. Legally, service allocators did not have the authority to delegate care tasks to family members. They stated that once a care recipient reached 18 years of age, family members were not legally obliged to provide care.

Service allocators put notable emphasis on the value of enabling older adults to remain at home with the support of their families. However, they explained that their ideal goal was to assess older adults’ needs without taking family members’ contributions into account. This preference arose from the municipality’s legal obligation to cover its population’s long-term care needs. However, it was standard practice for service allocators to collect information about the family members of older adults. One of the purposes of sourcing this information was to identify potential informal caregivers. This ambiguity in the roles of family members complicated the service allocators’ involvement efforts. One service allocator illustrated this ambiguity:

*We can’t take* [them] *into account… well*,* we do take* [the caring contributions of] *family members into account*,* but they don’t have any responsibilities. We have somehow become more concerned with that*,* thinking that if someone is coming home from a nursing home stay*,* they are coming home to an empty house. And what kinds of services should the municipality provide*,* and so on*,* to enable them to live at home? But we often include family members and the network in the assessment as well.* (M3, I8).

The service allocators explained that some family members expressed a desire to actively take part in caring for older adults, relieving the municipalities of a considerable portion of their caregiving duties. Service allocators expressed appreciation for family members’ active involvement in care delivery and included their contributions in the needs assessment. However, challenges sometimes arose due to issues of safety when family members were unable to provide care in accordance with professional standards of safety and/or dignity. In such cases, service allocators had to persuade family caregivers to withdraw from certain care tasks and accept more services. An illustrative example was a case where service allocators received a report of concern from home care providers about an older adult with amputated legs; family caregivers insisted on home living, but the service allocators advocated for nursing home placement due to safety concerns. If any adverse events occurred with these older adults, the municipality would legally bear the responsibility. Therefore, the voluntary involvement of family caregivers did not affect needs assessment in a way that led to a reduction in public services in these cases.

Conversely, in instances where service allocators assessed the situation as safe, home-dwelling older adults with substantial care needs could be denied services if they had support from family caregivers. This was especially the case in the assessment of applications for long-term nursing home placements. In these cases, having information about the family caregivers’ care contributions in the needs assessment was often crucial, as the safety of these older adults often depended on the support provided by their family members, who carried a substantial share of the care burden. Within these cases of transferred responsibility for covering older adults’ care needs, service allocators provided examples of daughters and sons who left their jobs or took sick leave due to their perceived personal responsibility for providing care for their parents. One example involved an older adult residing with his son. Despite the son’s concerns about leaving the father alone at home due to cognitive impairment and the amount of care needed, the application for a long-term nursing home stay was not approved. This example underscores the complexities of family involvement concerning older adults’ needs being voluntary or imposed on family members. When the needs assessment resulted in the rejection of a nursing home placement, the care responsibilities of family caregivers often increased. The service allocators explained that the service recipients in these cases often became subject to continuous safety assessments, in which the service allocators strove to keep the older adults at home as long as possible with the aid of family members and home care services. One service allocator elaborated:

*We have many* [older care recipients] *who are waiting for long-term nursing home placements*,* for example*,* who could not live at home if they didn’t have family members. And then suddenly we get a message of concern from the home care service that she was out walking last night*,* and then we see that it’s no good.* [Then, we ask the home care services: ] *Can you put in extra supervision tonight because we don’t have any* [nursing home places] *available now? We try continuously to balance this* [avoiding allocation of nursing home placements] *in relation to safety.* (M3, I7).

### Efficient involvement of family carers in needs assessment

In the process of assessing older adults’ care needs, service allocators strove for the efficient involvement of family members. Service allocators sought to allocate low-cost services due to a shortage of resources, and due to their limited time to gather information, they favoured the efficient involvement of family members, driven by their own needs for information exchange. Thus, family members were typically given a passive role in the allocation process and were typically involved in the needs assessment to capture family members’ perspectives on the care applicant’s care needs and home situation. Service allocators also worked proactively to ensure that family members had realistic expectations regarding the scope and necessity of the services allocated so that complaints and disagreements could be prevented.

When processing care applications from potentially new care recipients, service allocators invited family members to participate in home meetings to gather information, provide information and discuss the care applicant’s needs. Service allocators gained information about the presence of family members and how they wished to contribute and identified any limitations that family members encountered in meeting the older adult’s care needs. However, in several instances, service allocators did not communicate with family members during the needs assessment and allocation process. This was occasionally caused by family members not showing up at meetings or because the care applicant did not want family members present. Following the initial home visit, further dialogue with family members took various forms, including phone calls or, occasionally, discussions during a care recipient’s transition between the hospital or nursing home and their own home. However, it was more common for service allocators to be in dialogue with long-term care staff than the care recipient and their family members if the recipient was already receiving services, and direct communication was often initiated by family members. One service allocator explained:

*It is primarily the* [nursing home] *unit that maintains communication with family members. On rare occasions*,* if family members have decided to call me directly*,* I rarely say “No*,* you have to talk to* [someone else]*”*,* since we receive all kinds of calls. When family members call*,* they usually ask about service levels. It’s only when family members call and ask what kind of medication the patient should take at which times of the day*,* that I kind of refer: ‘Now you have to call the home care services and not me’. But otherwise*,* I do talk to family members.* (M1, I1).

The involvement of family members in needs assessment was highly regarded by service allocators due to the valuable information the family possessed about the care applicants. Consequently, the service allocation process was described as more efficient when family members were involved, as the needs assessment more accurately addressed the older adult’s care needs. Nevertheless, service allocators emphasised that the allocation of services was based on the assessment of care recipients’ needs, not how much family members fought for them. Despite this, service allocators acknowledged that the involvement of family members had the potential to influence the allocation process, particularly if the family provided new insights into the care needs of the recipient. However, in cases of disagreements between service allocators and family members regarding concerns and wishes, in most instances, this did not impact the service allocation outcome. For instance, disparities in viewpoints emerged when family members wanted several home visits per day for supervision purposes, while service allocators argued that a social alarm in the home was an adequate alternative. Service allocators explained that such disagreements highlighted the importance of providing clear information to family members about the extent to which older adults experiencing functional decline could safely live at home with the assistance of home services and welfare technology. One service allocator elaborated:

*Family members only see it from their perspective. They have only one mother*,* whom they love dearly and are very worried about.* […] *And they probably don’t work in the health care sector themselves. They might work as an accountant and have no idea how bad a state people actually can be in before they can’t live in their own homes anymore. They are not able to judge where the line goes for adequate and safe services. Because for them*,* this is the worst thing they have seen or the most worried they have been. So*,* just acknowledging it*,* calming them and telling them that we’ve got this* [is important]. (M1, I4).

### Support: preserving the family caregiver role

From the perspective of saving municipal resources, service allocators highlighted family members as valuable contributors to care delivery for older adults. Emphasising the importance of sustaining family caregiver resources, service allocators sometimes probed during the needs assessment about family caregivers’ health status and whether they were experiencing exhaustion or concerns. However, when caregivers needed support, family caregivers or long-term care staff usually initiated contact. The need for involving family caregivers in needs assessment was also discussed in the allocation meetings with long-term care staff in cases where care recipients had high care needs due to cognitive impairment or substantial functional decline or because they were receiving palliative care. In some of these cases, meetings with family caregivers were held to find solutions to meet the care needs of the older adults. This could, for example, result in the allocation of additional home care services to support family caregivers.

In conversations with family caregivers or long-term care staff, service allocators assessed caregivers’ needs for a break from their care responsibilities. The service allocators clarified that, according to legal requirements, they were obligated to allocate respite care to recipients whose family caregivers had substantial care responsibilities. These requirements for respite could be identified either during the initial home visit or through concerns raised by home care or nursing home staff or by the family caregivers contacting the service allocators. Respite, encompassing adult day-care services and short-term stays in nursing homes, was granted by service allocators when they observed that family caregivers were fatigued from or overwhelmed by extensive care responsibilities. One service allocator illustrated this with an ongoing case where the care recipient lived with his son and daughter-in-law:

*So*,* as long as the son and daughter-in-law have agreed to* [taking care of the father (-in-law)] *in exchange for getting all that relief*,* I think: ‘So great that that is possible!’ But then I also depend on them trusting me*,* and that I can say: ‘You know what*,* I’m going to reject the application* [for a long-term nursing home placement] *you submitted in summer because he’s not sick enough. Sorry. He is too well functioning. And he doesn’t want to be in a nursing home either. But I will give you relief. And the day things don’t work anymore*,* I’ll help you.’ At least we postpone the need* [for a nursing home placement]. *But the need will come. And then we must handle it before it becomes unsafe.* (M1, I4).

Demonstrating flexibility with family caregivers with extensive care responsibilities, service allocators actively strove to allocate respite services in a predictable manner. Upon identifying the need for respite, service allocators strategically allocated respite stays in nursing homes, for example, for 1–2 weeks four times a year. While addressing the needs of family members through predictable respite arrangements, service allocators also sought to postpone the need for the allocation of a long-term nursing home placement. The recipients who were eligible for the allocation of respite stays in a nursing home were individuals who would otherwise require long-term nursing home placement due to caregiver burnout. Thus, the services allocators’ consideration of family caregiver involvement was aimed at supporting them in their essential caregiver roles and preserving them as a care resource:

*After all*,* respite care is given to support family members who have a particularly heavy care load.* […] *They don’t get any healthier*,* care recipients who live at home*,* but family members may hang in there longer. Maybe you can postpone a long-term placement a little in that they have more predictable respite opportunities* […] *We have some* [care recipients] *whose supervision needs are very high*,* so it is admirable… There are many family members who are in very*,* very demanding situations every single day and over time. So*,* the more respite services and flexible respite options there are*,* the better it is for the municipality.* (M3, I8).

## Discussion

This study explored the involvement of family members in long-term care service allocation processes for older adults from the perspective of service allocators in Norway. The overarching theme in our findings was the struggle to balance family involvement concerning legal rights, efficiency, and welfare. Navigating legal obligations presents a challenge in reconciling the voluntary nature of the family caregiver role with the expectations service allocators have of family members’ caring contributions. Service allocators clearly acknowledge the importance of involving family members in the allocation process, although they give family members a predominantly passive role. Their primary purpose in involving family is to gather information and reconcile family members’ expectations of public services. It is more common for family members to initiate contact with service allocators than vice versa, and when family members contact service allocators, they usually have concerns and/or information about care recipients’ needs. Nonetheless, in many situations, service allocators view family members’ input as extremely valuable to needs assessment, particularly in situations where care recipients have substantial care requirements and family caregivers need respite.

Overall, this study shows that service allocators’ involvement of family members is restrained, primarily due to organisational boundaries, including resource scarcity and an information-oriented approach that hinders active involvement. Many service allocators clearly desire to involve family members in the assessment and allocation process. However, the gap between service allocators, care recipients and their family members has widened since the introduction of the purchaser-provider model [57]. As the findings in this study show, once a person receives services, the service allocators primarily contact long-term care staff for updates about changes in the care recipient’s care needs, thus reducing the need for direct contact with family members through the care trajectory. Across various OECD countries, there is a growing demand for public services to effectively integrate informal caregivers in care provision due to increasing demands on public care resources caused by population ageing [6]. Thus, at the national government level in Norway, there is a recognised need for enhanced collaboration between informal and formal long-term care [58]. Our findings show that the service allocation process is in accordance with ideas from NPM, where efficiency is central [27]; however, this leads to consequences such as less flexible services and reduced time for the involvement of service users and family members [57, 59–62]. The initial home visits to care applicants follow a standardised process, wherein close family members are invited and given the chance to participate. The engagement of family members, especially in the early phases of the care trajectory, appears arbitrary because, in several cases, family members do not attend these meetings.

Additionally, the involvement of family members is primarily characterised by information exchange, where information about the services is given to ensure realistic expectations. The care recipient is an integral part of the system receiving information, and both information-sharing and involvement should be based on the care recipients’ needs and preferences. This process may involve negotiations to reach a shared understanding of both needs and potential services among the health care professionals, the care recipients and the family members [12, 13]. Information exchange is considered to mean low involvement and participation of family members. Arnstein’s [63] conceptualisation of the “ladder of citizen participation” illustrates a continuum of citizen involvement, where ascending the ladder signifies an increase in the power of citizens over decision-making. Positioned at the lower levels are “informing” and “consultation”, where citizens are allowed to hear and have a voice but lack the power to ensure that their views are considered by those in power. This reflects the current role of family members in service allocation, where their voices may be acknowledged but does not translate into actual involvement. The higher levels of the ladder comprise “partnership” and “citizen control”, where citizens can negotiate and/or obtain full managerial power [63]. Applying this framework to family involvement in service allocation reveals that the current practice remains near the bottom of the ladder. Family members are rarely empowered as true partners in shaping the care process. Our results indicate that the involvement of family members in the allocation process is low and often initiated by family members and that family members are assigned a passive role. In dialogue with family members, service allocators explore how family members can contribute to meeting the care needs of older adults, aiming to avoid services. Additionally, one of the purposes of respite is to postpone the need for services, such as long-term nursing home placements. Thus, the reasons for involving family members are occasionally discussed in terms of improving efficiency rather than focusing on active involvement or the benefits for family members [64]. This efficiency-driven rationale aligns with a managerial paradigm that may conflict with higher levels on Arnstein’s ladder, where genuine partnership or citizen control is prioritised. The results of this study show that service allocators face challenges in balancing family involvement with the emphasis on efficiency in needs assessment, which can result in a lack of thorough examination of the genuine involvement opportunities and desires of family members. Thus, principles from NPM may influence their involvement agency, potentially prioritising system efficiency over service user-derived values [60, 65]. However, achieving the higher levels of involvement with “citizen control” or equal power between service allocators and family members is not possible, nor should it be a goal, as the service allocators, as the stewards of community goods, have many factors to consider in balancing public and private value. These factors include individual care recipients’ needs as well as local and public priorities and resources. However, movement towards the middle of Arnstein’s ladder—particularly towards partnership—offers a realistic and ethically sound path for improving family involvement without compromising the service allocators’ responsibilities. Nevertheless, our results highlight the potential to involve family members to a greater extent in the allocation process.

The findings in this study indicate that there is a blurred line between service allocators’ expectations of family members’ caring contributions and the municipality’s legal responsibility for care provision, in which family members’ decision to care for their older family members is voluntary. The unclear (legal) boundaries of caregiving responsibilities between family members and professional long-term care staff may result in both active and passive mistakes related to older adults’ safety at home [66]. These dynamics must also be understood within the broader context of shifting welfare state arrangements, in which the public sector appears to be gradually retreating from direct service provision, thereby placing increased responsibility on individuals and their families [9, 11]. In this light, service allocators are not only assessing needs and coordinating care but also mediating the boundaries between public provision and informal support. Results from this study show that this balancing act involves complex judgements concerning the extent to which family members are both willing and able to contribute to care without compromising either the care recipient’s safety or the caregivers’ own welfare. The risk of caregiver distress increases with increasing frailty in older adults [67]. Such distress can come from safety concerns when family members are unable to administer care in alignment with professional standards of safety and/or dignity and when service allocators depend on family caregivers’ contributions. Despite the potential care burden, many family caregivers view caregiving positively and find it meaningful [68, 69]. Nevertheless, as found in this study, the voluntary nature of caregiving is often constrained by obligations, social expectations, and economic constraints, leading a significant portion of caregivers to perceive it as excessively demanding [69]. Our findings indicate that service allocators, to a considerable degree, make decisions with the involvement of family caregivers in providing care for care recipients in the period preceding long-term nursing home placement. Ultimately, decisions not to allocate nursing home placements when applied for can effectively lead to an increased (involuntary) care burden for family members. However, the distribution of responsibilities between the formal care system and family members undergoes a significant shift when care recipients transition from their own home to nursing home facilities. According to a Norwegian study on resource utilisation, family members of persons with dementia provided ten times as much assistance than home services during the period immediately preceding moving to a nursing home [70]. Conversely, the involvement of family members in the care process may decrease for various reasons once the municipality assumes 24-hour responsibility in the nursing home setting [70]. Achieving a more balanced distribution of resources and enhancing collaboration between long-term care and family efforts both before and after the move are thus crucial to avoid caregiver burnout [71, 72] and ensure that family caregiving remains voluntary and safe. To achieve such goals, there is a need for a more family-oriented approach in the long-term care system, enabling the involvement of family members at an earlier stage in the care trajectory of older adults.

### Methodological considerations

By employing a qualitative methodology, this study explored service allocators’ experiences involving family members in the needs assessment and allocation of long-term care services to older adults. A methodological strength lies in the incorporation of diverse data sources, including individual interviews, focus group interviews, and direct observations, as a confirming strategy aimed at enhancing the study validity. The methodological integrity was further strengthened by the utilisation of interview guides during data collection [73], which ensured that procedural consistency was upheld during the interview phase and strengthened dependability [74, 75]. All respondents were provided with identical sets of open-ended questions, and standardised protocols were followed during both individual interviews and focus group discussions. Furthermore, the collaborative involvement of all authors in the analytical process enhanced the consistency of the findings and their dependability [76].

A limitation of this study is the absence of family carers’ perspectives, which, if included alongside those of service allocators, could have offered a more comprehensive and balanced understanding of family involvement in needs assessment and service allocation processes. The informants in this study were predominantly nurses, and all were women. While the study acknowledges potential benefits from the inclusion of male participants and a more diverse representation of professions, the composition aligns with the prevailing demographics within the service, where the nursing profession accounts for more than 80% of service allocators [46]. Consequently, we consider the sample to be a fair representation of the specific context under study. Although the study’s applicability to diverse contexts may be limited by variations in long-term care service settings across countries, the universal nature of health care professionals’ encounters with family caregivers suggests that the study findings are relevant to settings outside Norway.

## Conclusions

Service allocators highlight the importance of involving family members in needs assessment and allocation of long-term care services for older adults. However, family involvement is inhibited by legal and organisational boundaries, such as resource constraints and efficiency strategies employed by service allocators. Principles derived from NPM policies influence the agency of family member involvement in allocation decisions, causing service allocators to assign family members a passive role. Actively involving family members early and systematically is important if a more balanced distribution of care responsibilities between long-term care services and families is desired; this may ensure that family members’ care contributions remain voluntary and prevent caregiver burnout and unsafe situations for care recipients. In other words, if one of the goals of long-term care systems in the 21st century is to enhance and improve the resources inherent in family caregiving, there is a real need for more family-oriented approaches in long-term care, including in the allocation process. Further research is needed to explore methods for improving the involvement of family members in long-term care service allocation, planning and delivery.

## Supplementary Information

Supplementary Material 1.

Supplementary Material 2.

## Data Availability

No datasets were generated or analysed during the current study.

## Abbreviations

OECD: Organisation for Economic Cooperation and Development
NPM: New Public Management
M: Municipality
I: Informant
