# Supplementary material for: Contested involvement of family members in service allocation processes in long-term care: a qualitative study
Source: BMC Geriatr. 2026 Mar 3;26:476. doi: 10.1186/s12877-026-07265-5 (PMC13064338; doi:10.1186/s12877-026-07265-5)
Supplement: Supplementary file 1 — Supplementary Material 1. [file 12877_2026_7265_MOESM1_ESM.pdf]

Consolidated criteria for reporting qualitative research (COREQ): a 32-item checklist for interviews and focus groups

Domain 1: Research team and reflexivity

Personal Characteristics

1. Interviewer/facilitator. Which author/s conducted the interview or focus group?

In the "Methods" section, under the heading "The interviews".

2. Credentials. What were the researcher's credentials? E.g. PhD, MD

In the "Methods" section, under the heading "The interviews".

3. Occupation. What was their occupation at the time of the study?

In the "Methods" section, under the heading "The interviews".

4. Gender. Was the researcher male or female?

In the "Methods" section, under the heading "The interviews".

5. Experience and training. What experience or training did the researcher have?

In the "Methods" section, under the heading "The interviews".

Relationship with participants

6. Relationship established. Was a relationship established prior to study commencement?

In the "Methods" section, under the heading "Recruitment and participants".

7. Participant knowledge of the interviewer. What did the participants know about the researcher? e.g. personal goals, reasons for doing the research.

In the "Methods" section, under the under the heading "The interviews".

8. Interviewer characteristics. What characteristics were reported about the interviewer/facilitator? e.g. Bias, assumptions, reasons and interests in the research topic.

In the "Methods" section, under the heading "The interviews".

Domain 2: study design

Theoretical framework

9. Methodological orientation and Theory. What methodological orientation was stated to underpin the study? e.g. grounded theory, discourse analysis, ethnography, phenomenology, content analysis.

The methodology is described in the "Methods" section under the heading "Study design and setting" and under the heading "Coding and data analysis".

Participant selection

10. Sampling. How were participants selected? e.g. purposive, convenience, consecutive, snowball.

In the "Methods" section, under the heading "Recruitment and participants".

11. Method of approach. How were participants approached? e.g. face-to-face, telephone, mail, email.

In the "Methods" section, under the heading "Recruitment and participants".

12. Sample size. How many participants were in the study?

In the "Methods" section, under the heading "Recruitment and participants".

13. Non-participation. How many people refused to participate or dropped out? Reasons?

N/A

Setting

14. Setting of data collection Where was the data collected? e.g. home, clinic, workplace.

In the "Methods" section, under the heading "The interviews".

15. Presence of non-participants. Was anyone else present besides the participants and researchers?

In the "Methods" section, under the heading "The observations".

16. Description of sample. What are the important characteristics of the sample? e.g. demographic data, date.

In the "Methods" section, under the heading "Data".

Data collection

17. Interview guide. Were questions, prompts, guides provided by the authors? Was it pilot tested?

In the "Methods" section, under the heading "The interviews".

18. Repeat interviews. Were repeat interviews carried out? If yes, how many?

N/A

19. Audio/visual recording. Did the research use audio or visual recording to collect the data?

In the "Methods" section under the heading "Coding and data analysis".

20. Field notes. Were field notes made during and/or after the interview or focus group?

In the "Methods" section, under the heading "The observations".

21. Duration. What was the duration of the interviews or focus group?

In the "Methods" section, under the heading "The interviews".

22. Data saturation. Was data saturation discussed?

In the "Methods" section, under the heading "Recruitment and participants".

23. Transcripts returned. Were transcripts returned to participants for comment and/or correction?

N/A

Domain 3: analysis and findings

Data analysis

24. Number of data coders. How many data coders coded the data?

In the "Methods" section under the heading "Coding and data analysis".

25. Description of the coding tree. Did authors provide a description of the coding tree?

N/A

26. Derivation of themes. Were themes identified in advance or derived from the data?

In the "Methods" section under the heading "Coding and data analysis".

27. Software. What software, if applicable, was used to manage the data?

In the "Methods" section under the heading "Coding and data analysis".

28. Participant checking. Did participants provide feedback on the findings?

N/A

Reporting

29. Quotations presented. Were participant quotations presented to illustrate the themes / findings? Was each quotation identified? e.g. participant number.

Throughout the "Results" section and described in "List of abbreviations".

30. Data and findings consistent. Was there consistency between the data presented and the findings?

Yes.

31. Clarity of major themes. Were major themes clearly presented in the findings?

Yes.

32. Clarity of minor themes. Is there a description of diverse cases or discussion of minor themes?

Yes.
